# Status, causes and consequences of physicians’ self-perceived professional reputation damage in China: a cross-sectional survey

**DOI:** 10.1186/s12913-021-06306-6

**Published:** 2021-04-14

**Authors:** Tao Sun, Jinghui Wang, Shu’e Zhang, Yu Shi, Bei Liu, Xiaohe Wang

**Affiliations:** 1 Department of Health Management to School of Medicine, Hang Zhou Normal University, No.2318 Yuhangtang Road, Cangqian Street, Yuhang District, Hangzhou City, 311121 Zhejiang Province China; 2 College of Health Management of Harbin Medical University, Harbin, 150086 China; 3 Department of Epidemiology and Health Statistics, School of Public Health, Peking University, Beijing, 100191 China

**Keywords:** Chinese physicians, Deteriorating patient-provider relationship, Damaged professional reputation, Withdrawal behavior, Workplace well-being, Cross-sectional design

## Abstract

**Background:**

Conflict between physicians and patients is an increasingly serious problem, leading to the disrepute attached to Chinese physicians’ social image and position. This study assesses the status of physicians’ self-perceived professional reputation damage and explains it’s the adverse outcomes including withdrawal behavior and workplace well-being. Moreover, potential causes of Chinese physicians’ disrepute have been outlined.

**Methods:**

Primary data were collected through a cross-sectional online survey of physicians from 10 provinces in China, who were invited to complete an anonymous survey from December 2018 to January 2019. A total of 842 physicians (effective response rate: 92.22%) were recruited as participants.

**Results:**

About 83% of the participants self-perceived professional reputation damage from the sense of the public opinion concept. Approach half of participants exhibited the idea of turnover intention (47.3%) and one or more symptoms of burnout (46.4%). About 74.9% of the participants experienced a degree of stress. Additionally, three out of five participants reported low-level subjective well-being. More than 70% of the participants disapproved of their offspring becoming a physician. Four factors leading to physicians’ damaged professional reputations are those addressed: conflict transfer, cognitive bias, improper management, and individual deviance. Stigmatised physicians are more likely to practice high-frequent defensive medicine (*β* = 0.172, *P* <0.001), intend to leave the profession (*β* = 0.240, *P* <0.001), disapprove of their children becoming physicians (*β* = 0.332, *P*<0.001) and yield worse levels of workplace well-being, including high levels of perceived stress (*β* = 0.214, *P* <0.001), increasing burnout (*β* = 0.209, *P* <0.001), and declining sense of well-being *(β* = − 0.311, *P*<0.001).

**Conclusion:**

Chinese physicians were aware of damaged professional reputations from the sense of the public opinion concept, which contributes to increasing withdrawal behaviors and decreasing workplace well-being—a worsening trend threatening the entire health system. This novel evidence argues a proposal that Chinese health policy-makers and hospital administrators should promote the destigmatization of physicians immediately.

**Supplementary Information:**

The online version contains supplementary material available at 10.1186/s12913-021-06306-6.

## Background

Ten years ago, China launched a new national health system reform with the promise to provide universal, affordable, efficient, and equitable basic health care service for all its citizens by 2020 [1]. The reform’s first phase (2009–11) had given top priority to expanding social health insurance coverage for all and strengthening infrastructure. The second phase (2012 onwards) emphasised reforming its healthcare delivery system [2]. China has made substantial progress in improving equal access to care, delivering universal health coverage [3], and enhancing financial protection over the past decade [2]. As of end of 2018, China’s basic medical insurance and social old-age pension schemes has covered 1.35 billion people and more than 900 million people, respectively, together constituting the largest social safety net in the world [4]. Moreover, China is seeking to rebuild the primary health-care system [5], further establishing a tiered health-care delivery system to overhaul the existing hospital-centric approach [2]. Further, its central government emphatically and authoritatively declares that a principle of reform is ‘Person-Centred’ [6]. ‘Person-centred care’, defined by McCormack et al. [7], refers to ‘an approach to practice established through the formation and fostering of therapeutic relationships between all care providers, patients, and other individuals significant to them in their lives’. In this framework, all providers play an important role. More interestingly, most of the policy practices during the progress of health system reform seem to exhibit a uniform trend toward ‘Patient-Centred care’. Differentiation between two health care concepts of person- and patient-centred care has been clearly confirmed and discussed [8]. Seemingly, concern regarding medical staff, especially physicians, is completely excluded from current reforms. However, without an active participation of Chinese physicians and an improvement of their social and economic status, China’s health system reforms cannot be successful [9]. Physicians represent the major group in the delivery of health care services, but Chinese physicians are often victims in the new round of health system reform, facing the challenge of terrible violence [10], heavy workload, and an imbalance between effort and rewards [11]. Thus, growing attention has been focused on their unfavorable and awkward working environment [12], especially on deteriorating patient-provider relationships.

Min Zhou pointed out that four main issues, including funding shortage, an incompetent health insurance system, excessively market-oriented operation, and the media, jointly caused increasing patient–provider conflicts [13]. He further asserted that China has entered into the ‘dark ages’ with respect to patient–provider interaction [13]. Is it a sensational argument? It seems not to be; China’s patient-provider relationships have become increasingly serious in recent years, and Chinese physicians face a high risk of harm and common safety threats at the hospital workplace [10]. A national survey carried out by research team in 2017 revealed that physicians’ exposure to violence towards them is common in China; approximately 83.4% of participants had suffered more than one sort of violence [14]. Not only that, ‘Yi Nao’ (a Chinese term) is a unique issue in China, defined as gangs or individuals whose purpose is to force the hospital to reduce costs or to obtain compensation from hospitals, which has caused aggravating medical and hospital disturbances [15]. So far, the topic of violent incidents against medical staff and ‘Yi Nao’ has received extensive attention and became a source of controversy in China.

Workplace violence against physicians tend to jointly trigger a series of adverse effects on Chinese physicians, such as job dissatisfaction [16], low performance, perceived stress [17], job burnout [18], high turnover intention [16], sleep disturbance [17], poor mental health [19], and increased rate of medical error and reduced service quality [20], among others. These effects have been classified as psychological and emotional consequences that impact work functioning—main categories of outcomes arising from physicians’ exposure to workplace violence or poor patient-provider relationships [20]. However, this category seems to exclude cognitive elements, which have been widely ignored in previous studies. In fact, widespread national media reporting ‘bad medical news’, lacking objectivity, accelerates the destruction of the trust-based patient–provider relationship [21]. As a worse result, rising disputes between physicians and patients and related negative public opinion in regard to the health care industry is likely to evoke a cognitive bias in the whole society, in which physicians identify themselves as being hated and maligned by public. Moreover, their self-perception of their social reputations has worsened, particularly in today’s internet age. Indeed, comparing ancient and modern times, there has been a huge evolution in the perception of a physician’s role in China. Physicians are no longer on a pedestal in current society [22]. Chinese physicians’ incomes are low when compared with the high incomes paid in other industries that require less developed faculties or skills than medicine [23]. Public opinion that has maligned the physicians and poor compensation have bred fear among physicians [24], which contributes to physicians’ cognitive change regarding damaged self-perception and appraisal of their collective reputation and social position.

Considering that a physician’s social reputation is an enormously valuable asset, while the individual capability to manage his or her reputation is weak [25], self-perceived professional reputation damage among Chinese physicians tends to increase their lack of security, which results in other outcomes that have been given little attention. Previous research on reputation damage has focused mainly on special group of people with diseases such as AIDS [26], mental illness [27], tuberculosis [28], various cancers [29], obesity, diabetes, psoriasis, ADHD, epilepsy, and others. There are common similarities that these researches all discussed health-related stigmatization in patients. Indeed, reputation damage or stigmatization and its outcomes widely were investigated in patients with different kinds of diseases. The stigmatization regarding different groups is becoming a prevalent social problem in each country worldwide. Recently, extensive research has been conducted on public attitudes towards psychiatrists in Western societies [30]. In fact, people with higher social status more likely encounter common stigmatized reputation in current society, leading to an occupational stigma that staffs receive a negative reputation from public, which has caught academic attention. Moreover, reputation management strategies have been proposed in previous research [31]. However, little work has been performed on the self-perceived professional reputation damage of the physician group, which stands to benefit from an improvement in patient-provider relationship. Seemingly, this novel issue can’t get attention of Chinese health policy-makers and practitioner.

Due to lack social approval from patients and public, self-perceived professional reputation damage is a process by which a physician associates himself or herself with the disrepute of the vocation. The general attitudes of patients, their relatives, and public toward the physician group tend to influence the self-image and self-worth of members of the physician community in particular. There is not a single study focusing on self-perceived public attitudes toward Chinese physicians, and quantitative studies on physicians’ self-perceived professional reputation damage is especially scarce. Moreover, it is important to test whether the negative consequences of self-perceived professional reputation damage among Chinese physicians persist and cause adverse effects in decision-making behavior and subjective sense of workplace well-being.

Consequently, this study aims to (1) identify the prevalence and severity level of self-perceived professional reputation damage of Chinese physicians; (2) test the association between self-perceived professional reputation damage and withdrawal behavior and workplace well-being; (3) identify the reasons likely to contribute to the rising public negative evaluation attached to Chinese physicians based on the physicians’ own perspectives.

## Methods

### Subjects and procedures

An anonymous online questionnaire was completed by physicians across the country from December 2018 to January 2019 in China. The questionnaire was a cross-sectional survey involving 10 provinces. These physicians from Harbin Medical University were selected as ‘original participants’ of this survey, while the other participants included colleagues, classmates, or friends invited by them. A website link (https://www.wjx.cn/jq/31579963.aspx) about the questionnaire was sent to mobile phones of the subjects. A total of 913 participants were included in this survey, and ultimately 842 valid questionnaires were collated as analysis samples, with a 92.22% effective response rate.

The research described in this article meets the ethical guidelines of the ethics committee of the College of Public Health, Harbin Medical University. This project has been approved by the Ethics Committee of Harbin Medical University (ECHMU). Written informed consent cannot be procured because of the online and anonymous nature of the survey. Hence, oral informed consent for the survey was approved by the ECHMU and obtained from each physician. Before distributing the questionnaire, we formally informed every participant of the anonymity and privacy protection measures of the questionnaire. Dialogue and alert pop up boxes will appear on the screen of his/her mobile phone, which were used to remind participants who agreed to complete the survey to send their replies to our research group. Thus, once a questionnaire was completed, it was assumed that the physician orally agreed to participate in current survey by reference to Wen’s criteria [32].

### Measures

#### Measurement of physicians’ self-perceived professional reputation damage

Single-item measures, which are easy and simple to administer, were prevalently found to be useful in areas such as health and business [33]. In current study, a single-item measure of self-perceived professional reputation damage of physicians was: ‘In general, in your opinion, what is the attitude toward or evaluation on the Chinese physicians’ group among patients, their relatives, and the general public?’ Results were recorded on a 5-point Likert scale varying from 1‘very positive’ to 5 ‘very negative.’ For the sake of clearly addressing the degree of self-perceived professional reputation damage, the scores of ‘1’ and ‘2’ were recoded as ‘0’, which represents that sampled physicians have not perceived professional reputation damage from public. The scores of ‘3’, ‘4’, and ‘5’ were recoded as ‘1’, which indicates they perceived professional reputation damage from the general public.

#### Measurement of likely causes Lead to damaged reputation

A total of 32 items developed by the authors were used to measure the potential causes that led to patients’ or public’s negative impressions on Chinese physicians based on victims’ (i.e. physicians’) self-estimation. The response was recorded on a 5-point Likert scale varying from 1 ‘Strongly disagree’ to 5 ‘Strongly agree’. Four dimensions were identified, analysed, and presented in the Results chapter of this paper, including Conflict Transfer, Cognitive Bias, Improper Management and Individual Deviance.

#### Measurement of physicians’ withdrawal behaviors

Three items were used to measure “defensive medicine”, “turnover intention”, and “participants’ attitudes toward the idea of their offspring becoming a physician”. Three questions were jointly used to represent the physician’s withdrawal behavior. The question used to monitor defensive medicine was: ‘In the past year, do you practice defensive medicine to avoid medical risks or disputes during your routine clinical work?’ The response was recorded on a 5-point Likert scale varying from 1 ‘Never’ to 5 ‘Nearly all the time’. The question used to measure turnover intention was: ‘In the past year, did you have an intention to leave your current position?’ The response was recorded on a 5-point Likert scale varying from 1 ‘Never’ to 5 ‘Nearly all the time’. The following single-item measure of participants’ attitude toward the idea of their offspring becoming a physician was used: ‘What is your attitude towards the idea of your offspring becoming a physician in future?’. The response was recorded on a 5-point Likert scale varying from 1 ‘Strongly agree’ to 5 ‘Strongly disagree’.

#### Measurement of physicians’ workplace well-being

Three items in monitoring the “perceived stress”, “job burnout”, and “subjective sense of well-being” were selected to represent the workplace well-being of the sample physicians. A single-item measure of perceived stress as a more sensitive indicator of well-being in work organizational studies than illness-based health measures was developed by Elo A-L et al. [34]. This single-item measure of perceived stress had been proved to be a valid measure for drawing group-level conclusions about mental well-being [34]. The responses were recorded on a 5-point Likert scale varying from 1 ‘Not at all’ to 5 ‘Very much’. Moreover, a single-item burnout question as an alternative to the Maslach Burnout Inventory in order to abbreviate survey material and potentially increase response rates, developed by Rohland, B. M. et al. [35] was used to measure burnout symptoms among sampling physicians. Another item was used to measure subjective sense of life well-being of participations. We use a 5-point Likert scale: subjective sense of well-being: 1 = Very unhappy, 5 = Perfectly happy.

### Statistical analysis

Differences in continuous variables were examined using the one-way ANOVA. Pearson’s correlation coefficients were used to estimate correlations between the self-perceived professional reputation damage, withdrawn behavior, and workplace well-being. Hierarchical linear regression analysis was performed to test the effects of self-perceived professional reputation damage on these groups of dependent variables. The demographic variables related to self-perceived professional reputation damage in univariate analysis (*P* < 0.05) were entered into step 1 of the hierarchical regression analysis model in order to eliminate their interference with the dependent variables. We provided data including *F*, R^2^ and R^2^-changes, and the fit of the model was assessed with R^2^. A standardization regression coefficient (*β*) and *P* value for each step in the regression model were also produced. In this study, statistical significance was set at *P* < 0.05 (two-tailed). All of the above analyses were conducted using SPSS 24.0 for Windows.

## Results

### Demographic information for participants and their status of self-perceived professional reputation damage

Demographic variables of participants and the ANCOVA models for the self-perceived professional reputation damage of Chinese physicians were outlined in Table 1. A total of 699 (83%) participations thought that they had perceived that physicians to be a stigmatised group in the views of Chinese general population. Hence, current results indicated that Chinese physicians perceived their own severely damaged reputation.

**Table 1:** Characteristics of the Respondents (*n* = 842)

Characteristics	N	%	M ± SD	***F/t***	***P***
**Self-perceived professional reputation damage**	699	83%			
**Sex**					
Male	391	46.4	3.54 ± 1.05	***t =*** 10.352	< 0.001
Female	451	53.6	3.32 ± 0.94		
**Age**					
≤ 30	196	23.3	3.31 ± 0.92	***F =*** 1.548	0.201
31–40	328	39.0	3.45 ± 0.98		
41–50	220	26.1	3.50 ± 1.06		
≥ 51	98	11.6	3.37 ± 1.06		
**Service Years**					
≤ 2	206	24.5	3.32 ± 0.93	***F =*** 0.965	0.426
5–9	147	17.5	3.44 ± 1.03		
10–14	153	18.2	3.50 ± 0.95		
15–19	75	8.9	3.36 ± 0.82		
≥ 20	261	31.0	3.47 ± 1.10		
**Hospital level**					
Tertiary hospital	673	79.9	3.47 ± 1.00	***F =*** 2.741	0.042
Second-class hospital	131	15.6	3.22 ± 0.97		
Primary hospital	17	2.0	3.35 ± 1.00		
Non-classified hospital	21	2.5	3.14 ± 0.79		
**Education categories**					
College degree or below	37	4.4	2.81 ± 0.91	***F =*** 5.930	< 0.001
Bachelor	410	48.7	3.39 ± 1.02		
Master	310	36.8	3.50 ± 0.92		
Doctor	85	10.1	3.55 ± 1.12		
**Marital status**					
Single	183	21.7	3.27 ± 0.96	***F =*** 3.902	0.021
Married	627	74.5	3.48 ± 1.01		
Divorce or loss of spouse	32	3.8	3.19 ± 0.82		
**Professional position**					
Without professional title	55	6.5	3.09 ± 0.84	***F =*** 5.190	< 0.001
Resident physician	224	26.6	3.26 ± 0.97		
Attending physician	242	28.7	3.55 ± 0.98		
Associate chief physician	184	21.9	3.58 ± 0.99		
Chief Physician	137	16.3	3.38 ± 1.07		
**Monthly income**					
≤ 3000 Yuan	98	11.6	3.34 ± 0.93	***F =*** 0.643	0.632
3001–6000 Yuan	323	38.4	3.39 ± 1.00		
6001–9000 Yuan	238	28.3	3.50 ± 0.99		
9001–20,000 Yuan	157	18.6	3.43 ± 1.03		
≥ 20,000 Yuan	26	3.1	3.42 ± 1.06		

### Description results of Main variables

All description results of the main variables in this study are presented in Table 2, which shows that participants reported that the self-perceived professional reputation damage of Chinese physicians is caused by groups including patients, their relatives, and the public. Approximately 83% of participants perceived negative social impressions from the public. Approach 47.3% participants reported that they had the turnover intention. Additionally, more than 70% of the participants disagreed or strongly disagreed with the prospect of their offspring becoming a physician. About 74.9% participants experienced a degree of stress. Three out of five participants reported low-level subjective well-being. Near 46.4% participants reported definitely burning out and had one or more symptoms of burnout, such as physical and emotional exhaustion, even experienced symptoms of burnout and completely burned out.

**Table 2:** Description information of main variables (*n* = 842)

Level	n	%	accumulative percent
**Self-perceived professional reputation damage**			
Very Positive	13	1.5	1.5
Positive	130	15.4	17.0
General	334	39.7	56.7
Negative	219	26.0	82.7
Very Negative	146	17.3	100.0
**Defensive Medicine**			
Never	97	11.5	11.5
Very seldom	300	35.6	47.1
Quite often	286	34.0	81.1
Very often indeed	126	15.0	96.1
Nearly all the time	33	3.9	100.0
**Turnover Intention**			
Not at all	161	19.1	19.1
Very seldom	283	33.6	52.7
Sometimes	197	23.4	76.1
Often	140	16.6	92.8
Nearly all the time	61	7.2	100.0
**Career choice advice (the attitude toward his/her offspring becoming a physician)**			
Strongly Agree	12	1.4	1.4
Agree	112	13.3	14.7
Neutral	151	17.9	32.7
Disagree	320	38.0	70.7
Strongly Disagree	247	29.3	100.0
**Perceived Stress**			
Not at all	53	6.3	6.3
Not Often	158	18.8	25.1
Sometimes	320	38.0	63.1
Usually	234	27.8	90.9
Definitely	77	9.1	100.0
**Subjective Well-being**			
Very unhappy	37	4.4	4.4
Unhappy	152	18.1	22.4
Uncertainty	318	37.8	60.2
Happy	314	37.3	97.5
Perfectly happy	21	2.5	100.0
**Burnout**			
I enjoy my work. I have no symptoms of burnout.	22	2.6	2.6
Occasionally I am under stress, and I don’t always have as much energy as I once did, but I don’t feel burned out.	429	51.0	53.6
I am definitely burning out and have one or more symptoms of burnout, such as physical and emotional exhaustion.	202	24.0	77.6
The symptoms of burnout that I’m experiencing won’t go away. I think about frustration at work a lot.	126	15.0	92.5
I feel completely burned out and often wonder if I can go on. I am at the point where I may need some changes or may need to seek some sort of help.	63	7.5	100.0

### Exploratory factor analysis and reliability analysis of the causes leading to self-perceived professional reputation damage of Chinese physicians

Exploratory factor analysis was conducted to explore the structure of causes. Results reveal that the Bartlett spherical test coefficient is 0.000, representing an obvious level of significance. The KMO coefficient is 0.957, which is greater than 0.900. The Bartlett spherical test illustrates that there are several common factors among the 32 projects.

As a primary result of the principal component analysis, two items should be deleted owing to their two mutual factors. Ultimately, the principal component analysis suggested a 4-factor structure, accounting for 64.11% of all variances (21.86% by Factor 1, 16.90% by Factor 2, 14.06% by Factor 3, and 11.30% by Factor 4). The pattern and structures of the rotated common factors are shown in Table 3. The internal consistency reliability for the 30-item had an overall Cronbach’s α coefficient of 0.950. Moreover, the standardized factor loading of all the items ranges was above the threshold limit of 0.5 and above suggested by Hair et al. [36]. Internal consistency reliability analyses showed good internal consistency for all projects.

**Table 3:** Rotated factor loading matrix of all items

Items	Factor Loading
**Factor 1: Conflict transfer from the / an imperfect health system and changing society**	
Incompatible functions between health system and insurance, medical expenses are overburdening patients.	0.769
Government investment in healthcare industry is inadequate, while the reason for increasing medical expenses is often mistakenly attributed to Chinese physicians.	0.767
Imperfect salary structure in public hospitals results in a low reward of Chinese physicians.	0.765
The unclear boundary between the power and responsibility caused by a defective legal system result in increasing difficulty to maintain the interests of both physicians and patients in China.	0.749
Chinese physicians are overloaded owing to consulting with an excessive number of patients.	0.713
High-quality resources are concentrated in the big cities, resulting in overcrowding in large hospitals.	0.693
Current social trust is generally lower in China, which in turn exacerbates the gap in trust between the physicians and patients.	0.684
Information regarding the negative reputation of physicians diffuses quickly in an internet era of easy interconnection.	0.631
Public and media platforms are prone to ideologies of conspiracies and lack recognition for medical profession and clinicians.	0.623
During this social transformation period, wide-ranging inequity triggers public dissatisfaction with all Chinese industries.	0.558
**Factor 2: Individual Deviance Behavior of Physicians**	
Physicians’ Communication skill with patients is poor.	0.806
The service attitude of some physicians is not good.	0.803
The professionalism of some physicians is absent.	0.775
Busy physicians are without enough time to care for their patients.	0.770
Alert physicians often distrust their patients or their relatives.	0.695
Pursuing the economic interests of some physicians leads to occasionally excessive diagnosis and treatment.	0.688
Professional ability and skill Some physicians are deficient.	0.688
**Factor 3: Cognitive Bias of Social Public**	
Some patients or their relatives are hot-tempered and fail to cooperate with their physicians.	0.771
Some patients or their relatives are biased and show distrust toward Chinese physicians.	0.717
Social media used to promote reproachful narration toward Chinese physicians without professional or medical judgment.	0.693
Some patients or their relatives often raise unreasonable expectations of their physician.	0.683
Social media used to pursue to sensational press or reports regarding physician-patient dispute event lead to magnified, distorted, and amplified results.	0.647
The patients or their relatives believe that either the physician or hospitals generate the result of Chinese high medical expenses.	0.579
Chinese public hate to seek laws to deal with a medical error or accident.	0.577
**Factor 4: Improper Management in Public Hospital**	
Inadequate cooperation between different departments in Chinese public hospitals lends to low-efficiency management.	0.769
Due to poor procedure (awaiting long queues during many operations and steps such as registration, complications with receiving a diagnosis and getting medicine) patients’ time in consultation and treatment services is shortened.	0.695
Hospitals pay much attention to economic benefits, which in turn increase patients’ medical costs.	0.626
Hospital management is not scientific and chaotic.	0.614
Some hospitals’ medical equipment and hardware are inadequate.	0.611
The setting, operation, and medical institutions in public hospital are user-friendly.	0.587

### Correlations among study variables

The means, standard deviations, and Pearson correlation coefficients of continuous variables are shown in Table 4. As shown in the results, all variables were significantly correlated with each other. Self-perceived professional reputation damage was positively correlated with defensive medicine (*r* = 0.205, *P* < 0.01), turnover intention (*r* = 0.253, *P* < 0.01), career choice advice (*r* = 0.333, *P* < 0.01), perceived stress (*r* = 0.229, *P* < 0.01), and burnout (*r* = 0.236, *P* < 0.01) and was negatively correlated with subjective sense of well-being (*r* = − 0.343, *P* < 0.01).

**Table 4:** Means, standard deviation (SD) and correlations of continuous variables (*n* = 842)

Variables	Mean	SD	1	2	3	4	5	6	7
1. Self-perceived professional reputation damage	3.42	1.00	1						
2. Defensive medicine	2.64	1.00	0.205^**^	1					
3. Turnover intention	2.59	1.18	0.253^**^	0.220^**^	1				
4. Career choice advice	3.81	1.05	0.333^**^	0.082^*^	0.355^**^	1			
5. Perceived stress	3.15	1.03	0.229^**^	0.199^**^	0.467^**^	0.234^**^	1		
6. Burnout	2.74	1.00	0.236^**^	0.259^**^	0.537^**^	0.294^**^	0.551^**^	1	
7. Well-being	3.15	0.90	−0.343^**^	−0.208^**^	− 0.493^**^	− 0.340^**^	− 0.402^**^	− 0.466^**^	1

Note: ^**^ *p* < 0.01, Pearson Correlation is significant at the 0.01 level (2-tailed)

### Hierarchical linear regression models

Two groups of hierarchical regression analyses were performed to examine the influence of self-perceived professional reputation damage on withdrawal behavior (Table 5) and workplace well-being (Table 6), after eliminating the effects of demographic variables.

**Table 5:** Hierarchical linear regression models for withdrawal behaviors

Variables	Defensive medicine		Turnover intention		Career choice advice	
	*M*_*1*_*(β)*	*M*_*2*_*(β)*	*M*_*3*_*(β)*	*M*_*4*_*(β)*	*M*_*5*_*(β)*	*M*_*6*_*(β)*
**Control variables**						
Age	0.117	0.129	−0.083	− 0.067	− 0.119	− 0.096
Sex	− 0.242	− 0.224^**^	−0.013	0.012	0.011	0.046
Marital status	−0.023	−0.028	0.015	0.007	−0.010	−0.021
Service Years	0.079	0.073	0.079	0.071	−0.007	−0.019
Hospital level	−0.012	0.000	0.058	0.075^*^	−0.026	−0.003
Education level	0.048	0.035	−0.038	−0.057	− 0.049	−0.075^*^
Professional title	−0.050	−0.065	0.077	0.055	0.117	0.087
Monthly income	−0.052	−0.025	− 0.173^***^	−0.134^***^	− 0.127^*^	−0.074
**Independent variable**						
Self-perceived professional reputation damage		0.172^***^		0.240^***^		0.332^**^
*F*	9.471^***^	11.552^***^	4.646^***^	9.940^***^	2.486^*^	13.443^***^
*R*^*2*^	0.083^***^	0.111^***^	0.043^***^	0.097^***^	0.023^*^	0.127^***^
*△R*^*2*^	0.083^***^	0.028^***^	0.043^***^	0.054^***^	0.023^*^	0.104^***^

*Notes*-*M*_*1,*_ *M*_*3,*_*M*_*5*_: the influence of demographic variables on the Defensive medicine, Turnover intention and Career choice advice; *M*_*2,*_*M*_*4,*_*M*_*6*_: the influence of Self-perceived professional reputation damage on on the Defensive medicine, Turnover intention and Career choice advice; *Notes*-hierarchical linear regression ^*^ *p* < 0.05 (2-tailed), ^**^ *p* < 0.01 (2-tailed), ^***^ *p* < 0.001 (2-tailed)

**Table 6:** Hierarchical linear regression models for workplace well-being

Variables	Perceived stress		Burnout		Well-being	
	*M*_*7*_*(β)*	*M*_*8*_*(β)*	*M*_*9*_*(β)*	*M*_*10*_*(β)*	*M*_*11*_*(β)*	*M*_*12*_*(β)*
**Control variables**						
Age	−0.114	−0.100	−0.016	−0.002	0.089	0.068
Sex	−0.072^*^	−0.050	− 0.118^**^	−0.096^**^	0.125^***^	0.092^**^
Marital status	0.094^*^	0.087^*^	0.037	0.030	0.030	0.040
Service Years	0.022	0.015	−0.050	−0.058	0.011	0.022
Hospital level	0.025	0.040	−0.020	−0.006	− 0.012	−0.033
Education level	−0.009	−0.026	0.026	0.010	0.011	0.035
Professional title	0.057	0.038	0.028	0.009	−0.179^**^	− 0.151^*^
Monthly income	−0.106^**^	− 0.072^*^	−0.131^***^	− 0.098^**^	0.179^***^	0.130^***^
**Independent variable**						
Self-perceived professional reputation damage		0.214^***^		0.209^***^		−0.311^***^
*F*	2.703^**^	6.776^***^	3.726^***^	7.549^***^	6.442^***^	16.201^***^
*R*^*2*^	0.025^**^	0.068^***^	0.035^***^	0.075^***^	0.058^***^	0.149^***^
*△R*^*2*^	0.025^**^	0.043^***^	0.035^***^	0.041^***^	0.058^***^	0.091^***^

*Notes*-*M*_*7,*_ *M*_*9,*_*M*_*11*_: the influence of demographic variables on the Perceived stress, Burnout and Well-being; *M*_*8,*_*M*_*10,*_*M*_*12*_: the influence of Self-perceived professional reputation damage on the Perceived stress, Burnout and Well-being; *Notes*-hierarchical linear regression ^*^ *p* < 0.05 (2-tailed), ^**^ *p* < 0.01 2-tailed), ^***^ *p* < 0.001 (2-tailed)

As shown in Table 5, self-perceived professional reputation damage was positively associated with defensive medicine (*β* = 0.172, *P* <0.001). These results are also revealed in Table 5. We find that self-perceived professional reputation damage was positively associated with turnover intention (*β* = 0.240, *P* <0.001). Moreover, a positive correlation between self-perceived professional reputation damage and career choice advice (i.e. as parents, Chinese physicians disapprove of their offspring becoming a physician when given a choice of occupation) was observed in the subjects (*β* = 0.332, *P* <0.001). The results suggest that self-perceived professional reputation damage of Chinese physicians was positively correlated with their withdrawal behaviors. So, the high level of physicians’ self-perceived professional reputation damage is closely related to negative organizational behaviors and aversion to the occupation of a physician.

Correlation between self-perceived professional reputation damage among Chinese physicians and their workplace well-being also were tested, and all results are presented in Table 6. These results indicate that three hierarchical linear regression analyses all exhibited statistical significance. We found that self-perceived professional reputation damage was positively associated with perceived stress (*β* = 0.214, *P* <0.001) and job burnout (*β* = 0.209, *P* <0.001) and was negative associated with subjective sense of well-being (*β* = − 0.311, *P* <0.001). Therefore, the high level of physicians’ self-perceived professional reputation damage is negatively related to workplace well-being.

## Discussion

### Chinese physicians were aware of severe self-perceived professional reputation damage

This study investigated the status of physicians’ self-perceived professional reputation damage and its effect on several facets of organizational behavior and workplace well-being. A national study witnessed that the deteriorated patient-provider relationship has serious ramifications for physicians’ public and self-perception in China [37]. Similarly, we further found that Chinese physicians have suffered widely from disrepute and stigmatization. About 83% of participants perceived professional reputation damage or estimation from patients, private citizens, and the general public. Chinese physicians were subjected to a powerful stereotype by patients, who consider the physicians’ profession rife with avaricious, indifferent, selfish, and insidious individuals. Unsurprisingly, there are widespread conflicts between physicians and patients, which further raise concerns about adverse narratives in the media and negative public opinion toward Chinese physicians, especially regarding their behavior and level of professionalism. Indeed, health care in China still faces huge challenges [38], such as a poor capacity of the primary health system, increasing expenditure, insufficient and incompetent personnel, inefficient use of resources, and other issues. As potential causes, the factors under study jointly exacerbated various conflicts between physicians and patients, as proven by current sample physicians. China is undergoing huge social change caused by rapid economic growth, which leads to the universal social conflicts existing in China today [32]. Thus, as an extension of the conflict, issues such as the distrust between people and disagreement concerning benefits between public and individual physicians are often present in the medical industry. In fact, there are some physicians with a low level of professionalism who exhibit individually deviant behavior [39], such as receiving ‘red-envelope money’, engaging in bribery, receiving kickbacks, and showcasing bad attitudes and lack of humanity in medicine [40]. Moreover, improper operations also exist in hospital management settings, including inadequate hospital institution, low efficiency of service, long queues for awaiting registration, lack of empathy, unreasonable bonus schemes [41], as well as profit-driven operation, management and performance assessment [37].

To avoid magnifying influence, hospital managers and health policy-makers used to compensate patients who engaged in ‘YI NAO’, even in the case where physicians or hospitals were without an accident. As a result, hospitals’ response strategies to ‘YI NAO’ often motivate other patients and their families to follow unexpectedly. The increasing occurrence of ‘YI NAO’ has created negative opinions about Chinese public hospitals and physicians among the public and media. In addition, there is significant cognitive bias against Chinese physicians among patients and the general public, who allege that physicians do not master enough medical information of patients, and often are driven by economic interest, which contributes to a distrust of physicians’ medical decision-making. Therefore, four chief factors (conflict transfer, cognitive bias, improper management, and individual deviance) were reported by Chinese physicians by current survey as reasons leading to physician’s damaged professional reputation. To some extent, this study not only gives comprehensive answers regarding the degree of self-perceived professional reputation damage among Chinese physicians, it can also serve as a proposal to destigmatize the profession and promote the reform of China’s health system. An honest statement of fact about social phenomenon of ‘YI NAO’ in China is beyond the scope of current study. Moreover, it must be mentioned that potential biasness present in the current study due to the collection way of the data collected by using the online resources were prone to obtain the evidence was rather limited.

Additionally, the current study also indicated that approach a half of participants exhibited the idea of turnover intention and one or more symptoms of burnout. More serious, about 74.9% participants experienced a degree of stress. Moreover, three out of five participants reported low-level subjective well-being. Above these findings are similar conclusions with other report’s [42], which together imply that Chinese physicians are suffering from the increasingly serious occupational environment and awful well-being. In this case, more than 70% of the participants disapproved of their offspring becoming a physician, which also is a novel finding from current study. This worsening trend is threatening the entire health system, especially to physician personnel, which needs to catch the attention of health policy-makers in China.

### Physicians’ self-perceived professional reputation damage was positively associated to withdrawn behavior

We further found the significant associations between physicians’ self-perceived professional reputation damage and withdrawn behavior and workplace well-being. Current findings revealed that self-stigmatized physicians are more likely to practice high-frequent defensive medicine, greater turnover intention, and more likely disapprove of offspring becoming a physician.

Physicians’ self-perceived professional reputation damage results in a high risk for themselves in regard to interactions between physician and patient, which further contributes to a curious phenomenon that physicians being faced with a number of challenges when using the quality of patient-provider relationship as a toolkit for decision-making [43]. Furthermore, when some patients are regarded as potential troublemakers who are likely to present a high risk of ‘Yi Nao,’ more defensive medicine will be practiced by physicians toward them to protect themselves avoiding being attacked [44]. Undoubtedly, the social reputation and status of physicians group are gradually weakened in China, which tends to present the healthcare industry as unattractive, further increasing turnover among physicians. Most importantly, current survey supported the fact that new generations of Chinese physicians are likely to face a crisis [45]. Additionally, these physicians had reported that they regretted entering the medical profession and attempted to leave it [46]. The current study also observed that physicians’ experience of damaged professional reputation led them to identify their career as negative and prevented them from encouraging the next generation to become physicians, hampering recruitment of young physicians among medical graduates [47]. Based on analysis results, 38.0% and 29.3% of participants disagree and strongly disagree, respectively, with the prospect of their offspring becoming physicians. Similarly, another more serious phenomenon needs to be concerned that since physician suicide was a significant problem, i.e., male doctors had suicide rates as much as 40% higher than the general population, female doctors up to 130% higher [48], and that cross-sectional findings demonstrated higher rates of depression in medical trainees compared to the general population [49], which seems to be ignored due to systemic barriers that discourage self-care and help-seeking behaviors among physicians. This shocking discovery should stimulate China’s central government to pay more attention to Chinese physicians’ salaries, social status, workplace well-being, and dignity. Those offensive or prejudicial assessments on Chinese health profession and industry as a social stereotype are hurting physicians’ well-being and health. On the one hand, future studies should pay attention to explore the rates of psychiatric disorders, e.g., anxiety, depression, and suicidality among physicians and if they actually sought professional assistance. On the other hand, a positive, respectful and friendly professional reputation toward Chinese physician community is urgent for building a better health system, which has been neglected severely and slipped out the opinion of health policy-makers and practitioners.

### Self-perceived professional reputation damage was negatively associated to workplace well-being of Chinese physicians

This study outlined that stigmatized physicians experienced a lower level of workplace well-being, such as high perceived stress, increasing burnout, and decline in the subjective sense of well-being.

Stigmatized physicians were troubled by the distrustful relationship between physicians and patients in China, which led them to devote more energy to deal with interpersonal communication with patients and their families, representing a psychological expense and loss of psychological resources owing to the loss (or risk of loss) of patients’ trust and dignity. The risk of a physicians’ loss of professional reputation is a source of pressure. Admittedly, Chinese patient-provider relationship has historically been poor, which causes increasing physicians’ damaged reputation, perceived stress, and burnout. Thus, we can further argue that there are positive associations between tense patient-provider relationship, self-perceived professional reputation damage, perceived stress, and burnout. Similarly, subjective sense of well-being of stigmatised physicians certainly will be compromised. Overall, physicians’ self-perceived professional reputation damage was associated with low workplace well-being, which emphasizes a suggestion for health policy-makers and practitioners that maintaining physicians’ positive social image is a benefit for accelerating their health and wellness.

### Limitations

Some limitations must be acknowledged. First, a convenience sample was used in this study, which increased the potential for sampling bias. Second, a major limitation of the study is the cross-sectional design, whose nature cannot effectively and precisely judge the causal relationship between the variables. One important direction for future research involves longitudinal studies. Third, the data were collected from self-reports from physicians by means of an online survey, which is likely to introduce response bias from social desirability or negative affection. The physicians might have overestimated or underestimated the association between the study’s variables. Moreover, several single-item tools in this study were used for collecting the data in order to abbreviate survey material and potentially increase response rates, but which potentially reduce the validity and reliability of this measurement; therefore, a widely used measure tool should be adopted in the future.

## Conclusions

The current study enhances the knowledge by investigating the status of self-perceived professional reputation damage of Chinese physicians and its association with withdrawal behaviors and workplace well-being.

Physicians with a high level of self-perceived professional reputation damage were more likely to practice defensive medicine from avoiding the risk of suffering medical dispute. This study broadens the understanding of the nature of the association between patient-provider relationship and defensive behaviors. Physicians practice defensively because of self-perceived threats caused by self-perceived professional reputation damage arising from tense physician- patient relationships.

Physicians who experience a high level of self-perceived professional reputation damage are more likely to quit or disapprove of their offspring becoming physicians. Meanwhile, current study already demonstrated that Chinese physicians universally were suffering from high-intention turnover, high-level stress, great-risk burnout, low-level subjective well-being, and were unreluctant to support their offspring becoming a physician. These findings are a meaningful reminder for policymakers to formulate policies to encourage qualified personnel to enter the Chinese health system.

Four potential reasons for physicians’ damaged professional reputation were clarified by using an exploratory factor analysis, which includes conflict transfer, cognitive bias, improper management, and individual deviance. This category contributes to the preliminary literature by supplying a better understanding of potential reasons for conflicts between physicians and patients, especially as these conflicts relate to the topic of health system reform in China.

In addition, a negative association between self-perceived professional reputation damage of Chinese physicians and workplace well-being was illustrated, which demonstrated that physicians with a high level of self-perceived professional reputation damage also suffer greater perceived stress and burnout and a reduced subjective sense of well-being. This finding indirectly indicates that poor patient-provider relationships threaten physicians’ mental health and well-being through physicians’ increasing self-self-perceived professional reputation damage.

Finally, we advocate for interventions to reduce physicians’ damaged professional reputation, which will, directly or indirectly, protect physicians’ benefits and well-being. Physicians’ professional reputation management strategies should be proposed in future. A favourable public opinion environment towards the medical industry and its health care staff will contribute to improved patient-provider relationships, which will promote effective health system reform.

## Supplementary Information

**Additional file 1.**

## Data Availability

Data is currently not available online, but the data can be obtained upon request from hydsuntao@126.com.

## Abbreviations

AIDS: Acquired Immune Deficiency Syndrome
ADHD: Attention Deficit Hyperactivity Disorder
ECHMU: Ethics Committee of Harbin Medical University
ANCOVA: Analysis of Covariance
KMO: Kaiser-Meyer-Olkin
M: Mean
SD: Standard Deviation
