# Supplementary material for: Status, causes and consequences of physicians’ self-perceived professional reputation damage in China: a cross-sectional survey
Source: BMC Health Serv Res. 2021 Apr 14;21:344. doi: 10.1186/s12913-021-06306-6 (PMC8048359; doi:10.1186/s12913-021-06306-6)
Supplement: Supplementary file 1 — Additional file 1. [file 12913_2021_6306_MOESM1_ESM.docx]

**A. Self-perceived Professional Reputation Damage**

| In general, in your opinion, what is the attitude toward or evaluation on the Chinese physicians’ group among patients, their relatives, and the general public? |  | - Very positive |
| --- | --- | --- |
|  |  | - Positive |
|  |  | - General |
|  |  | - Negative |
|  |  | - Very negative |

**B. Causes Lead to Chinese physicians’ Damaged Reputation**

**Please indicate to what extent you agree or disagree with the following statements.**

|  |  | strongly disagree | disagree | neutral | agree | strongly agree |
| --- | --- | --- | --- | --- | --- | --- |
|  |  |  |  |  |  |  |
| **B1** | Incompatible functions between health system and insurance, medical expenses are overburdening patients |  |  |  |  |  |
|  |  |  |  |  |  |  |
| **B2** | Government investment in healthcare industry is inadequate, while the reason for increasing medical expenses is often mistakenly attributed to Chinese physicians |  |  |  |  |  |
|  |  |  |  |  |  |  |
| **B3** | Imperfect salary structure in public hospitals results in a low reward of Chinese physicians |  |  |  |  |  |
|  |  |  |  |  |  |  |
| **B4** | The unclear boundary between the power and responsibility caused by a defective legal system result in increasing difficulty to maintain the interests of both physicians and patients in China |  |  |  |  |  |
|  |  |  |  |  |  |  |
| **B5** | Chinese physicians are overloaded owing to consulting with an excessive number of patients |  |  |  |  |  |
|  |  |  |  |  |  |  |
| **B6** | High-quality resources are concentrated in the big cities, resulting in overcrowding in large hospitals |  |  |  |  |  |
|  |  |  |  |  |  |  |
| **B7** | Current social trust is generally lower in China, which in turn exacerbates the gap in trust between the physicians and patients |  |  |  |  |  |
|  |  |  |  |  |  |  |
| **B8** | Information regarding the negative [reputation](http://dict.youdao.com/w/reputation/#keyfrom=E2Ctranslation) of physicians diffuses quickly in an internet era of easy interconnection |  |  |  |  |  |
|  |  |  |  |  |  |  |
| **B9** | Public and media platforms are prone to ideologies of conspiracies and lack recognition for medical profession and clinicians |  |  |  |  |  |
|  |  |  |  |  |  |  |
| **B10** | During this social transformation period, wide-ranging inequity triggers public dissatisfaction with all Chinese industries |  |  |  |  |  |
|  |  |  |  |  |  |  |
| **B11** | Physicians’ Communication skill with patients is poor |  |  |  |  |  |
|  |  |  |  |  |  |  |
| **B12** | The service attitude of some physicians is not good |  |  |  |  |  |
|  |  |  |  |  |  |  |
| **B13** | The professionalism of some physicians is absent |  |  |  |  |  |
|  |  |  |  |  |  |  |
| **B14** | Busy physicians are without enough time to care for their patients |  |  |  |  |  |
|  |  |  |  |  |  |  |
| **B15** | Alert physicians often distrust their patients or their [relatives](http://dict.youdao.com/w/relatives/#keyfrom=E2Ctranslation) |  |  |  |  |  |
|  |  |  |  |  |  |  |
| **B 16** | Pursuing the economic interests of some physicians leads to occasionally excessive diagnosis and treatment |  |  |  |  |  |
|  |  |  |  |  |  |  |
| **B17** | Professional ability and skill Some physicians are deficient |  |  |  |  |  |
|  |  |  |  |  |  |  |
| **B18** | Some patients or their [relatives](http://dict.youdao.com/w/relatives/#keyfrom=E2Ctranslation) are hot-tempered and fail to cooperate with their physicians |  |  |  |  |  |
|  |  |  |  |  |  |  |
| **B 19** | Some patients or their [relatives](http://dict.youdao.com/w/relatives/#keyfrom=E2Ctranslation) are biased and show distrust toward Chinese physicians |  |  |  |  |  |
|  |  |  |  |  |  |  |
| **B20** | Social media used to promote reproachful narration toward Chinese physicians without professional or medical judgment |  |  |  |  |  |
|  |  |  |  |  |  |  |
| **B21** | Some patients or their [relatives](http://dict.youdao.com/w/relatives/#keyfrom=E2Ctranslation) often raise unreasonable expectations of their physician |  |  |  |  |  |
|  |  |  |  |  |  |  |
| **B22** | Social media used to pursue to sensational press or reports regarding physician-patient dispute event lead to magnified, distorted, and amplified results |  |  |  |  |  |
|  |  |  |  |  |  |  |
| **B23** | The patients or their [relatives](http://dict.youdao.com/w/relatives/#keyfrom=E2Ctranslation) believe that either the physician or hospitals generate the result of Chinese high medical expenses |  |  |  |  |  |
|  |  |  |  |  |  |  |
| **B24** | Chinese public hate to seek laws to deal with a medical error or accident |  |  |  |  |  |
|  |  |  |  |  |  |  |
| **B25** | Inadequate cooperation between different departments in Chinese public hospitals lends to low-efficiency management |  |  |  |  |  |

| **B26** | Due to poor procedure (awaiting long queues during many operations and steps such as registration, complications with receiving a diagnosis and getting medicine) patients’ time in consultation and treatment services is shortened |  |  |  |  |  |
| --- | --- | --- | --- | --- | --- | --- |
|  |  |  |  |  |  |  |
| **B27** | Hospitals pay much attention to economic benefits, which in turn increase patients’ medical costs |  |  |  |  |  |

| **B28** | Hospital management is not scientific and chaotic |  |  |  |  |  |
| --- | --- | --- | --- | --- | --- | --- |
|  |  |  |  |  |  |  |
| **B29** | Some hospitals’ medical equipment and hardware are inadequate |  |  |  |  |  |

| **B30** | The setting, operation, and medical institutions in public hospital are user-friendly |  |  |  |  |  |
| --- | --- | --- | --- | --- | --- | --- |

**C. Withdrawal Behaviors**

| **C1** | In the past year, do you practice defensive medicine to avoid medical risks or disputes during your routine clinical work? |  | - Never |
| --- | --- | --- | --- |
|  |  |  | - Very seldom |
|  |  |  | - Quite often |
|  |  |  | - Very often indeed |
|  |  |  | - Nearly all the time |
|  |  |  |  |
| **C2** | In the past year, did you have an intention to leave your current position? |  | - Not at all |
|  |  |  | - Very seldom |
|  |  |  | - Sometimes |
|  |  |  | - Often |
|  |  |  | - Nearly all the time |
|  |  |  |  |
| **C3** | What is your attitude towards the idea of your offspring becoming a physician in future? |  | - Strongly agree |
|  |  |  | - Agree |
|  |  |  | - Neutral |
|  |  |  | - Disagree |
|  |  |  | - Strongly disagree |

**D. Workplace Well-being**

| **D1** | Stress means a situation in which a person feels tense, restless, nervous or anxious or is unable to sleep at night because his/her mind is troubled all the time. Do you feel this kind of stress these days? |  | - Not at all |
| --- | --- | --- | --- |
|  |  |  | - Very seldom |
|  |  |  | - Sometimes |
|  |  |  | - Often |
|  |  |  | - Very much |

**D2 Please estimates your level of burnout by following the definition of burnout.**

- I enjoy my work. I have no symptoms of burnout.
- Occasionally I am under stress, and I don’t always have as much energy as I once did, but I don’t feel burned out.
- I am definitely burning out and have one or more symptoms of burnout, such as physical and emotional exhaustion.
- The symptoms of burnout that I’m experiencing won’t go away. I think about frustration at work a lot.
- I feel completely burned out and often wonder if I can go on. I am at the point where I may need some changes or may need to seek some sort of help.

| **D3** | In the past week, what extent do you agree with following statement? |  | - Very unhappy |
| --- | --- | --- | --- |
|  |  |  | - Unhappy |
|  |  |  | - Neutral |
|  |  |  | - Happy |
|  |  |  | - Perfectly happy |

**E. Demographic characteristics**

| **E1** | What is your sex? |  | - Male |
| --- | --- | --- | --- |
|  |  |  | - Female |
|  |  |  |  |
| **E2** | What is your age? |  | - ≤30 years |
|  |  |  | - 31-40 years |
|  |  |  | - 40-50 years |
|  |  |  | - ≥51 years |
|  |  |  |  |
| **E3** | What is your service year? |  | - ≤2 years |
|  |  |  | - 5-9 years |
|  |  |  | - 14-14 years |
|  |  |  | - 15-19 years |
|  |  |  | - ≥20 years |
|  |  |  |  |
| **E4** | What is your hospital level? |  | - Tertiary hospital |
|  |  |  | - Second-class hospital |
|  |  |  | - Primary hospital |
|  |  |  | - Non-classified hospital |
|  |  |  |  |
| **E5** | What is your education category? |  | - College degree or below |
|  |  |  | - Bachelor |
|  |  |  | - Master |
|  |  |  | - Physician |
|  |  |  |  |
| **E6** | What is your marital status? |  | - Single status |
|  |  |  | - Married status |
|  |  |  | - Divorce or loss of spouse status |
|  |  |  |  |
| **E7** | What is your professional position? |  | - Without professional title |
|  |  |  | - Resident physician |
|  |  |  | - Attending physician |
|  |  |  | - Associate chief physician |
|  |  |  | - Chief Physician |
|  |  |  |  |
| **E8** | What is your monthly income? |  | - ≤3000 Yuan |
|  |  |  | - 3001-6000 Yuan |
|  |  |  | - 6001-9000 Yuan |
|  |  |  | - 9001-20,000 Yuan |
|  |  |  | - ≥20,000 Yuan |
|  |  |  |  |

**End of questionnaire, thank you for your co-operation!**
